# Lipid Peroxidation Produces a Diverse Mixture of Saturated and Unsaturated Aldehydes in Exhaled Breath That Can Serve as Biomarkers of Lung Cancer—A Review

**DOI:** 10.3390/metabo12060561

**Published:** 2022-06-18

**Authors:** Saurin R. Sutaria, Sadakatali S. Gori, James D. Morris, Zhenzhen Xie, Xiao-An Fu, Michael H. Nantz

**Affiliations:** 1 Department of Chemistry, University of Louisville, Louisville, KY 40292, USA; srsuta01@louisville.edu; 2 Department of Neurology, School of Medicine, Johns Hopkins University, Baltimore, MD 21205, USA; sgori3@jhmi.edu; 3 Department of Chemical Engineering, University of Louisville, Louisville, KY 40292, USA; james.morris.3@louisville.edu (J.D.M.); zhenzhen.xie@louisville.edu (Z.X.)

**Keywords:** lung cancer, exhaled breath, lipid peroxidation, aldehyde, unsaturated, biomarker, VOC, breath analysis

## Abstract

The peroxidation of unsaturated fatty acids is a widely recognized metabolic process that creates a complex mixture of volatile organic compounds including aldehydes. Elevated levels of reactive oxygen species in cancer cells promote random lipid peroxidation, which leads to a variety of aldehydes. In the case of lung cancer, many of these volatile aldehydes are exhaled and are of interest as potential markers of the disease. Relevant studies reporting aldehydes in the exhaled breath of lung cancer patients were collected for this review by searching the PubMed and SciFinder^n^ databases until 25 May 2022. Information on breath test results, including the biomarker collection, preconcentration, and quantification methods, was extracted and tabulated. Overall, 44 studies were included spanning a period of 34 years. The data show that, as a class, aldehydes are significantly elevated in the breath of lung cancer patients at all stages of the disease relative to healthy control subjects. The type of aldehyde detected and/or deemed to be a biomarker is highly dependent on the method of exhaled breath sampling and analysis. Unsaturated aldehydes, detected primarily when derivatized during preconcentration, are underrepresented as biomarkers given that they are also likely products of lipid peroxidation. Pentanal, hexanal, and heptanal were the most reported aldehydes in studies of exhaled breath from lung cancer patients.

## 1. Introduction

Perturbations of oxidant levels, such as reactive oxygen species (ROS), in the cellular matrix arise due to endogenous or xenobiotic processes that are either a cause or effect of various disease states. The oxidative stress (OS) related to such perturbations has been extensively studied and reported in the literature [1,2,3,4,5]. Redox imbalance is directly implicated in lung carcinogenesis; in particular, oxidative cleavage of lung tissue lipids is attributed either to systematic ROS or their presence in the lung organs [6]. Lipid peroxidation (LPO) is a widely accepted free radical process used to describe the oxidative destruction of unsaturated fatty acids. The products of such peroxidation have been confirmed in various biological matrices using a variety of techniques [7]. The wide interest in LPO-derived metabolites is due in part to the potential of these small, volatile organic products to serve as indicators of early-stage lung cancer [8].

Lung cancer (LC) is the most common form of malignancy in the world. A reported 142,080 people died from LC in the United States in 2018 [9]. The advent of computed tomography (CT) scanning has allowed for the large-scale screening for lung cancer. The surveillance and early diagnosis of LC leads to timely treatment and higher survival rates [10]. However, the disadvantages of repeated exposure to radiation from CT or positron emission tomography (PET) scans, the high false positive rates associated with this primary modality of LC screening, and the need for subsequent, more invasive technologies to confirm diagnoses have limited the wider application of these tests. It is therefore imperative to evaluate alternative methods of LC detection. One promising alternate modality is to analyze the exhaled breath of patients to detect products of LPO and thereby diagnose the presence, extent, and possibly even the type of LC [11].

While the existence of volatile LPO products in exhaled breath have been known since the 1970s, reports differ in terms of assigning the origin of the volatilome, the mechanism of oxidative breakdown, and the predictive value of exhaled markers for the clinical diagnosis of disease [12]. We have therefore examined the reports that correlate exhaled volatile organic compounds (VOCs) with incidences of LC. This study is aimed to investigate the reports of LPO products while also providing an explanation for the existence and predictive efficiency of individual markers in breath as they relate to lung cancer detection. Although systemic LPO is well established in the literature [13], our focus in the present review was to evaluate the aldehyde volatilome originating in the lungs. Other LPO-derived VOC families, such as alkanes, alkenes, and alcohols, have been discussed elsewhere and are not addressed in this review [14,15]. Limiting our study to aldehydes allowed us to focus on the particular species of markers that is expected in higher concentrations in exhaled breath, namely volatile aldehydes generated from the oxidative cleavage of mono-, di- and polyunsaturated lipids, which constitute nearly 69% of all phosphatidylcholines—the major lipid class in lung tissue [16]. The LPO products generated within the lungs are expected to better survive the endogenous environment compared to those originating systemically because they can rapidly exchange across the liquid–air interface within the alveoli as they are generated and then be exhaled.

To enumerate the source and predict potential aldehyde candidates of LPO due to lung disease, we followed a systematic approach:

(a) identify common unsaturated fatty acids found in lung tissue;
(b) based on the free radical mechanism of LPO, simulate oxidative cleavage of the identified panel of unsaturated lipids;
(c) list potential aldehyde products of LPO generated by the simulation;
(d) conduct a literature search for reports of the LPO products in exhaled breath and document the analytical techniques used to detect them.

## 2. Lipid Composition of Lung Tissue

To understand the origin of exhaled LPO products, it is first important to understand the source of these oxidative byproducts—the lipidome. The lipid composition of lung tissue is well understood [17]. The human lung is composed of a variety of cell types, and each cellular membrane contains a signature combination and high percentage of phospholipids [17]. The variations in phospholipids arise from a multitude of possible configurations involving different polar headgroups and types of fatty acid (FA) hydrophobic domains. FAs are incorporated in the cellular membranes and may be either saturated, monounsaturated, or polyunsaturated depending on the biochemical pathway activated for their de novo synthesis. The activation of such pathways may be triggered in response to endogenous or xenobiotic stimulus and may vary widely between individuals. Consequently, the resulting biosynthesis of lipids and the incorporated FAs vary accordingly. While there are abundant studies on the activation of biochemical pathways that result in the selective, enzymatic incorporation of specific FAs in the phospholipid framework, it is outside the scope of this review. We focused on the most common FAs reported in the lung tissue for the further evaluation of their respective LPO products [18].

**Figure 1:**
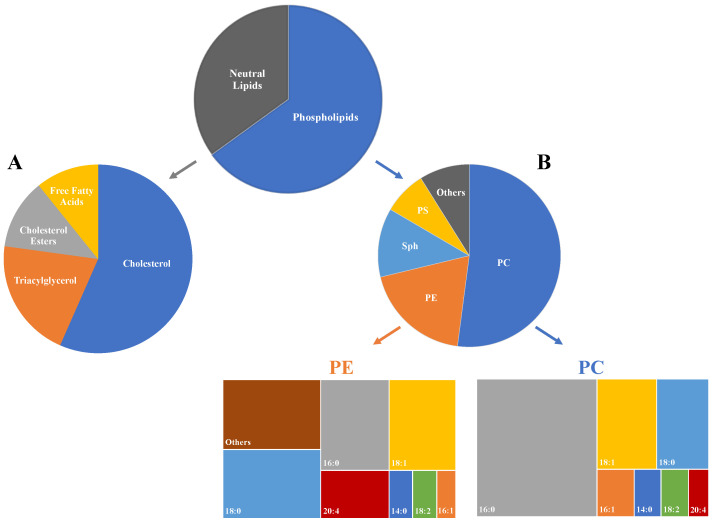
Lipid composition of lung tissue. (**A**) Neutral lipid breakdown; (**B**) phospholipid breakdown and relative fatty acid compositions (treemap charts) for the major phosphatides phosphatidylethanolamine (PE) and phosphatidylcholine (PC). For a key to abbreviations, see list at end of article.

Unlike other organs, lung epithelium (alveolar type II cells) also secretes a surfactant, composed mainly of lipids (90%) and protein (10%), that lines the surface and promotes alveolar stability by lowering surface tension [16]. Lung surfactant may be further classified based on its physical form; namely, tubular myelin, a monolayer at the air-liquid interface, or micellar lipid form. The levels of fatty acids in surfactant are also in a state of constant flux and vary from person to person. Lung surfactant has no unique phospholipids (PL); however, the combinations of fatty acids vary extensively. The PL composition of surfactant is more than three-quarters phosphatidylcholine (PC), and half of these PC lipids are polyunsaturated [19]. Due to the fact that excess ROS also react with surfactant lipids, leading to a weakened surface tension and the breakdown of lung surfactant as well as LPO products [20], the composition of surfactant lipids will have a bearing on the aldehyde volatilome. The commonly reported FAs found in human lung tissue and lung surfactant are provided in Table 1 [18], and the breakdown of lipids comprising lung tissue is shown in Figure 1 [16].

To determine the potential aldehyde volatilome that could be generated as a result of LPO in lung tissue, we applied the free radical oxidative cleavage mechanism of LPO to the relevant FAs (Table 1) as described in the next section [14,22]. This simulation generated a number of saturated aldehydes, α,β-unsaturated aldehydes, and hydroxyaldehydes. A thorough review of the literature for these compounds then led us to further classify the LPO-derived aldehydes as those that were either detected in exhaled breath or those that were subjected to discriminatory analyses and deemed to be indicators—biomarkers—of lung cancer.

## 3. Lipid Peroxidation

**Figure 2:**
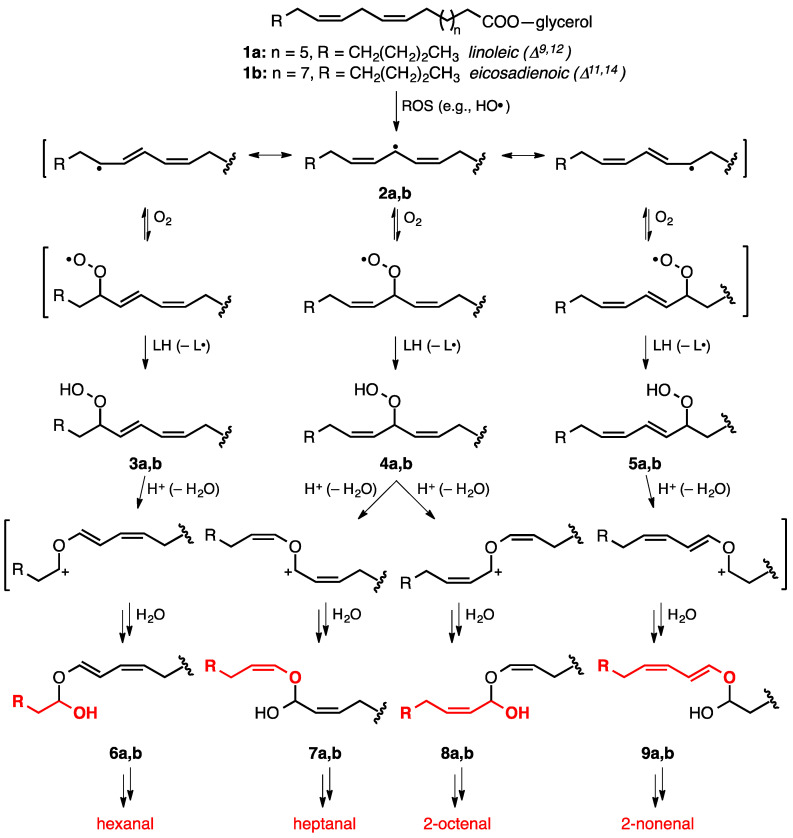
Polyunsaturated fatty acyl sidechain oxidation via free radical-mediated hydro-peroxide formation and decomposition leads to mixtures of saturated and unsaturated aldehydes (ROS = reactive oxygen species; LH = neighboring lipid). Atoms in red give rise to the aldehydes generated on hemiacetal equilibrium.

To understand the random mixture of aldehydes that is formed under LPO conditions, consider the representative reactions of the ω-6 fatty acids linoleic and eicosadienoic, **1a** and **1b**, respectively (Figure 2). While 2–5% of major pulmonary phospholipids are linoleic [23], eicosadienoic is not as prevalent but still common [24]. For reasons related to free radical stability [25], hydrogen atom abstraction by ROS generated under oxidative stress predominantly occurs at the bis-allylic methylene position to produce the doubly resonance-stabilized free radical **2**. Resonance delocalization leads to the scrambling of alkene stereochemistry to afford isomeric mixtures of *E* and *Z* alkenes at carbons 9–12 for **2a** and carbons 11–14 for **2b**. The subsequent reaction with molecular oxygen generates peroxyl radicals, which afford corresponding pentadienylic hydroperoxides, such as **3a**,**b** and **5a**,**b**, as well as bis-allylic hydroperoxides [26], such as **4a**,**b**, on hydrogen atom transfer from resident hydrogen donors that include neighboring PUFAs [27], resulting in the propagation of the free radical-mediated process.

The peroxyl radicals derived from PUFAs with high degrees of unsaturation often undergo cyclization reactions to generate cyclic peroxides [28], leading to complex mixtures on subsequent cleavage [29]. Redox active metals, such as Fe(II) [30], V(IV) or V(V) [31], and Cu(I) [32], deplete hydroperoxides by generating alkoxy radicals [33,34,35] that undergo carbon–carbon bond scission to release corresponding aldehyde and alkyl radical products [36]. Conversely, hydroperoxide activation via enzyme-mediated (e.g., phospholipid hydroperoxide glutathione peroxidase (phGPx) [37,38], cytochromes P450 (CYP2S1, CYP3A4) [39]) or acid-induced processes actuate Hock–Criegee rearrangements [40,41]—peroxide O–O cleavage via neighboring group 1,2-migration—to deliver the mixture of hemiacetals **6**-**9**. Each hemiacetal dissociates to two aldehydes, with the lipid tail-derived fragments (in red, Figure 1) producing the more volatile aldehyde fraction consisting of hexanal, heptanal, 2-octenal, and 2-nonenal. In the case of nonenal, the unsaturated aldehyde may be formed with β,γ-unsaturation that subsequently undergoes isomerization to the thermodynamically preferred α,β-position or react with ROS to produce hydroxylated products (e.g., 4-hydroxy-2-nonenal, 4-HNE), as discussed next [22].

**Figure 3:**
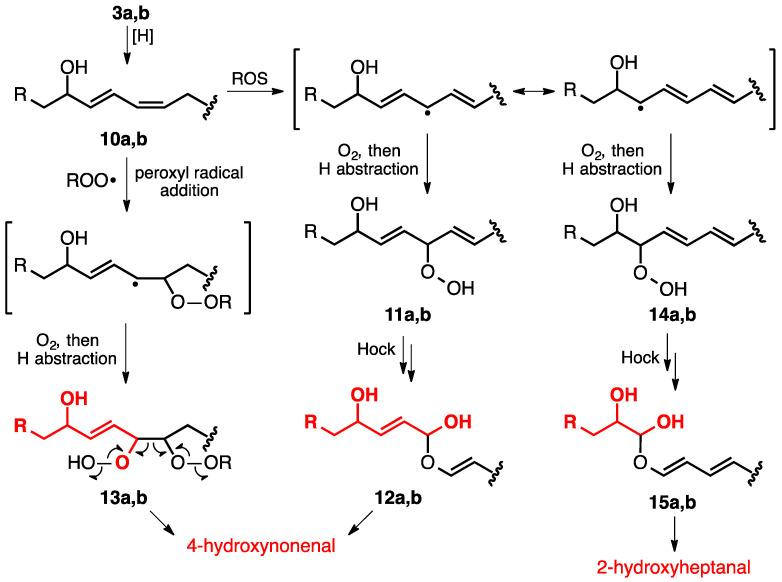
Bis-peroxidation pathways leading to the formation of 2- and 4-hydroxyaldehydes. Atoms in red give rise to the aldehydes generated by the indicated processes.

In addition to aldehydes derived from lipid mono-peroxidation, bis-peroxidation processes also contribute to diversify the mixture of volatile aldehydes generated under LPO conditions [22]. As examples, a reduction in lipid hydroperoxides **3a**,**b** delivers dienyl alcohols **10a**,**b** that undergo subsequent radical-mediated reactions with diatomic oxygen at various positions to afford hydroperoxides **11a**,**b** and **14a**,**b** after hydrogen atom transfers (Figure 3). These adducts then are transformed as noted above via hemiacetals **12a**,**b** and **15a**,**b** into the hydroxyaldehyde products 4-hydroxynonenal (4-HNE) and 2-hydroxyheptanal, respectively. Peroxyl radical additions to dienyl intermediates, such as **10a**,**b**, also occur to generate new radical species that can react with oxygen, as exemplified by the formation of **13a**,**b**. The subsequent fragmentation [42] of the 1,2-bishydroperoxides produces 4-HNE.

As shown in Figure 3, a principal 2-hydroxyaldehyde formed under oxidative stress is 2-hydroxyheptanal [43,44]. Whereas short-chain 2-hydroxyaldehydes are known as products of lipid peroxidation [43,45], detecting them in exhaled breath presents a considerable challenge due to their facile dimerization, a consequence of the higher carbonyl reactivity imparted by the inductive effect of the adjacent C-OH group [46]. α,β-Unsaturated aldehydes, such as 2-octenal, 2-nonenal, and 4-HNE (aldehydes formed in Figure 2 and Figure 3), are also more reactive than saturated counterparts, as they are electrophilic at both the carbonyl carbon and the β-carbon and able to undergo both 1,2- and 1,4-addition reactions [47]. Unsaturated aldehydes react with nucleophilic moieties of proteins and nucleic acids, modifying those molecules and effecting their function [36]. Substitution at the γ-carbon (4-position), as in 4-HNE and 4-hydroxy-2-hexenal (4-HHE), somewhat diminishes 1,4-addition reactivity due to steric and electronic considerations and thus confers a longer lifetime [48]. Indeed, 4-HNE is among the most detected and studied LPO products [49,50].

Figure 2 and Figure 3 illustrate the mixture of aldehydes and aldehyde types that can be formed under LPO conditions. The application of this process to a wider, representative selection of ω-3, -6, -7, and -9 unsaturated FAs taken from Table 1 creates a diverse panel of aldehydes, listed in Table 2, a result of the random nature of LPO. Efforts that have experimentally mimicked LPO conditions in vitro on MUFAs and PUFAs report many of these aldehydes. Tamura et al. carried out oxidations of mono- and polyunsaturated fatty acids with Fe(II) and hydrogen peroxide at 37 °C and found all but two of the α,β-unsaturated aldehydes listed in Table 2 [51]. In 2007, Kawai et al. reported 33 aldehyde products from in vitro lipid peroxidations at pH 7.4 and 37 °C matching four of the seven α,β-unsaturated aldehydes and four of the five hydroxyaldehydes listed in Table 2 [52].

Not shown in Table 2 are several low-molecular weight (C1–C3) aldehydes arising from lipid over-oxidation, secondary aldehyde oxidations, or amino acid metabolism; these include formaldehyde, acetaldehyde, hydroxyacetaldehyde, propenal (acrolein), and malondialdehyde (MDA). With the exception of MDA, these aldehydes are common in breath, often as a result of alcohol and tobacco use [53,54], and as such are not reliable as biomarkers of lung cancer. MDA, however, is a product of LPO and a well-established [55] marker of OS and will be discussed in a later section. Hydroxyacetaldehyde, a reported marker of lung cancer [56], is more closely linked to serine metabolism than lipid peroxidation [57,58].

## 4. Search Method and Results

Literature searches for the putative lung LPO-derived aldehydes were performed using the SciFinder^n^ and PubMed^®^ databases, last searched 25 May 2022, with no restrictions on date of publication. The searches used combinations of the keywords and phrases: lung cancer, breath, and marker. The PubMed^®^ searches had no exclusions, while SciFinder^n^ marked 70,178 reports to be ineligible using an automation tool due to a low relevance to the searched terms. Titles and abstracts were screened for reports of exhaled breath related to lung cancer. Those that passed the initial screening process and reported aldehydes were collected for this review. Table 3 is a summary of the results. Specifically, 16,378 records were screened by the author SRS, 114 were assessed for eligibility, and 44 studies spanning 34 years, from 1988–2022, were selected. Tabulated data from these 44 reports was reviewed by the authors ZX and JDM. Given the timespan of the reports in our study and differences in patient details reported, our analysis of the data does not include patient age, sex, or race. Smoking history, reported by some studies but not all, is also not tabulated in our analysis and is another limitation of this review.

## 5. Aldehydes Observed in the Exhaled Breath of Cancer Patients

The large variations in the reported median concentrations of the exhaled aldehydes in Table 3 are common and can be attributed to differences in VOC capture technology, particularly with respect to the different solid-phase microextraction (SPME) materials that were used (e.g., Carboxen-polydimethylsiloxane (CAR/PDMS) or divinylbenzene-Carboxen-PDMS (DVB/CAR/ PDMS) vs. Tenax extraction). Differences in SPME fiber exposure times, differences in desorption protocols and analysis processes, differences in the patient populations examined, especially with respect to LC staging, and differences in the type of lung cancer studied (non-small cell lung cancer (NSCLC) vs. small cell lung cancer (SCLC)) also contributed to widening the concentration ranges noted for aldehyde biomarkers [101]. Biomarker quantification is further complicated by the nature of the VOC mixture. Brunton et al. compared the adsorptions of aldehydes to different fibers and found that aldehyde recovery by Carbowax/DVB fiber, for example, was lowered by a factor of seven when exposed to an aldehyde mixture compared to recovery on exposure to singular aldehydes [102]. Concentration variations in a population of samples can also be due to environmental effects. Exogenous sources of aldehydes include food consumption, tobacco use, and even inhaled aldehydes from aging building and floor materials in indoor environments [6,103], thus requiring careful control measurements.

**Figure 4:**
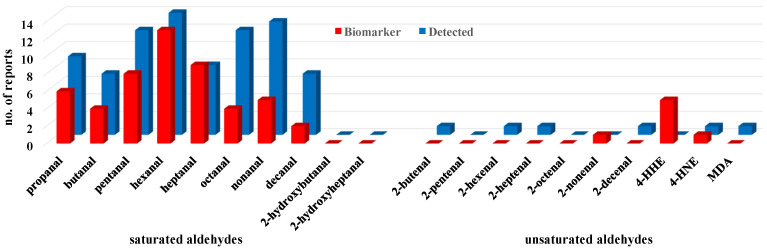
Number of literature reports for each aldehyde that was either detected (blue) in the exhaled breath of lung cancer patients or deemed a biomarker (red) of lung cancer. The term biomarker denotes a statistically significant increase in the EB of LC patients compared to healthy controls. Total reports for a given aldehyde are the sum of the red and blue columns.

From Table 3, when plotting both the incidence of aldehyde detection in lung cancer patient breath and when the presence of a given aldehyde was determined to be a biomarker of lung cancer for that study, a qualitative assessment of LPO-derived aldehydes as indicators of lung cancer becomes evident (Figure 4). Saturated aldehydes are particularly well represented, and their presence is often significantly different in the EB of LC patients relative to healthy control (HC) subjects (Figure 4, red bars). In contrast, hydroxyaldehydes and unsaturated aldehydes, which are derived from the same lipids and random LPO processes, are not widely observed, possibly a result of their higher intrinsic reactivity resulting in lower, trace concentrations in exhaled breath. Below are study details for the aldehydes summarized in Table 3.

## 6. Saturated Aldehydes

### 6.1. Propanal

The LPO source of propanal is ω-3 FAs. Six independent studies reported that propanal is significantly elevated in the EB of the LC patients relative to levels in HCs and smokers. Kischkel et al. reported a median concentration of propanal in LC patients of 0.34 nmol/L [68]. In comparison, the median propanal levels in HCs and smokers were both reported as 0.00 nmol/L. Poli et al. noted significantly higher levels of propanal relative to HCs, with mean concentrations of 0.054 nmol/L and 0.031 nmol/L, respectively [8]. Schallschmidt et al. reported a median propanal concentration of 1.01 nmol/L in LC patients and significantly lower levels of propanal in HCs [83]. Ulanowska et al. also observed higher levels of propanal in LC patients, reporting an average propanal concentration of 7.8 ppb, while measuring lower levels of propanal in the breath of HCs at an average concentration of 6.9 ppb [70]. Shehada et al. analyzed breath using silicon nanowire field effect transistors and identified propanal as a biomarker [85]. In 2021, Li et al. reported propanal as a biomarker with a significant increase in its concentration when comparing the breath of LC patients to that of HCs using a non-traditional on-paper derivatization SPME coupled with GC-MS analysis [95]. Similarly, Ligor et al. concluded that propanal was elevated in the EB of LC patients but did not claim that propanal could serve as a LC biomarker [80]. Rudnicka et al. (2011) reported propanal in the EB of LC patients having a concentration range of 0.66–3.74 ppb but not as a marker of LC [69]. In summary, propanal was reported in 34% of the studies collected for this review, and 40% of these studies determined propanal is a biomarker of LC. These investigations suggest that elevated levels of propanal may indeed be indicative of an underlying disease. However, one issue that complicates using propanal as a biomarker of cancer is its presence in ambient air [104], tobacco smoke [105], food [106], and other exogenous sources, such as car exhaust [107,108].

### 6.2. Butanal

The formation of butanal via LPO is restricted to the oxidation of ω-3 fatty acids. Several studies have measured butanal in EB [67]. Buszewski et al. measured butanal levels in LC patients at 1.32–2.55 ppb relative to concentrations in HCs at 1.35–1.87 ppb [72]. Similarly, Rudnicka et al. found butanal in EB at concentrations in the range of 0.78–2.55 ppb [69]. Kischkel et al. found the median butanal concentration of 1.81 nmol/L to be higher in LC patients only relative to the levels observed in smokers, while they noted no significant difference in comparison to levels in HCs, who presented higher median concentrations of butanal than LC patients [68]. In contrast, Poli et al. [8] observed butanal to be a reliable marker of NSCLC, and the mean butanal concentration was measured at 0.026 nmol/L compared to the mean level measured in HCs at 0.011 nmol/L. Schallschmidt et al. found butanal to be significantly elevated in the EB of LC patients, with a median level 0.014 nmol/L relative to a median level in HCs of 0.007 nmol/L [83]. Li et al. also recently reported butanal to be a biomarker of LC [95]. Similar to the challenge of using propanal as a biomarker, the numerous exogenous sources of butanal complicate the characterization of butanal as a biomarker. Common ambient butanal sources include tobacco smoke [109] and food [110]. Butanal is a principal VOC emitted from municipal solid waste treatment plants [111]. Whereas butanal was reported in only 25% of the studies reviewed, it was determined as a biomarker in 36% of those cases.

### 6.3. Pentanal

Pentanal is generated from ω-6 FAs. It was reported in 45% of the studies collected for this review, of which 40% noted significantly elevated levels in the EB of LC patients relative to HCs, making pentanal the second most reported aldehyde in this review. Fu et al. investigated both SCLC and NSCLC patients in comparison to patients with benign pulmonary nodules and HCs and found significantly higher pentanal levels only in SCLC patients [56]. In a follow-up study, the same group noted a statistically significant difference in pentanal levels between HCs, patients with benign pulmonary nodules, and those with LC, who had the highest levels of pentanal, with concentration thresholds ranging from 1.1–1.315 nmol/L [79]. Fuchs et al. observed the median pentanal concentration in LC patients to be 0.019 nmol/L relative to median levels in both HCs and smokers at 0.002 and 0.000 nmol/L, respectively [67]. Poli et al. reported a mean pentanal concentration in the breath of NSCLC patients of 19.1 pM in comparison to a mean concentration in HCs of 7.6 pM [8]. Ulanowska et al. measured the average concentration of pentanal in LC patients at 5.9 ppb and found that the HCs, which included healthy smokers, non-smokers, and past smokers, had an average concentration of 0.0 ppb [70]. Gashimova et al. reported the pentanal/acetonitrile ratio as a biomarker [94]. Three other groups reported pentanal as a biomarker of LC: Bajtarevic et al. in 2009 [64], Shehada et al. in 2016 [85], and Li et al. in 2021 [95]. Based on the differences in disease and control groups noted in these studies, there is good evidence that pentanal appears to be a breath biomarker of LC.

### 6.4. Hexanal

The LPO of both ω-6 and ω-7 FAs can lead to the formation of hexanal. It is the most widely reported LPO-derived aldehyde. Of the 44 reports collected for this review, 61% detected hexanal, of which 48% determined that hexanal is a biomarker of LC. Hexanal was observed using every reported technique of preconcentration and analysis method. Phillips et al. were the first to label hexanal as a biomarker of LC in EB in 1999 [60]. Fuchs et al. reported hexanal as an LC biomarker with an LC patient median concentration of 0.010 nmol/L compared to a HC median concentration at 0.00 nmol/L [67]. Ulanowska et al. determined hexanal as a biomarker with an average LC patient concentration of 4.5 ppb, also compared to a HC average concentration of 0.0 ppb [70]. Poli et al. reported hexanal in LC patients with a mean concentration of 0.037 nmol/L, significantly higher than the levels in HCs at 0.009 nmol/L [8]. Poli’s findings were cited and corroborated by Li et al. in finding hexanal to be an EB biomarker of LC [95]. Deng et al. found hexanal to be in the EB of LC patients but not in the EB of HCs [61]. Handa et al. deemed hexanal a biomarker; their report was one of the few not to use a preconcentration method and the only report using ion mobility spectrometry to detect VOCs [76]. In 2005, Chen et al. reported hexanal as a biomarker and were the only report to use a novel GC-SAW sensor for analysis [62]. In 2007, Chen et al. highlighted the VOCs present in the headspace, and in another, much larger, study with 160 LC patients in 2021, found hexanal to once again rise to the level of a biomarker of LC [93]. Rudnicka et al. [77], Gashimova et al. [94], and Zou et al. [100] also reported hexanal to be an EB biomarker of LC. Whereas Kischkel et al. measured hexanal to have a greater median concentration in the EB of LC patients (0.59 nmol/L) than that of healthy smokers (0.31 nmol/L), the concentration in LC patients was less than the median concentration they measured in HCs (0.63 nmol/L) [68]. Despite a few potential exogenous sources of hexanal [103,112], the number of reports detecting hexanal clearly suggest hexanal must be considered when evaluating for EB biomarkers of LC. Chen et al. reported that the headspace VOCs of stage I and II lung tumor tissue are the same as those in the headspace from stage III and IV lung tumor tissue. They found that hexanal is one of the headspace VOCs and subsequently determined hexanal to be a biomarker in the EB of LC patients [63].

### 6.5. Heptanal

Heptanal is a possible LPO product of both ω-6 and ω-7 FAs. It is the second most reported biomarker of LC from the papers collected for this study. Phillips et al. was the first to identify heptanal in exhaled breath as a biomarker of LC in 1999 [60]. Chen et al. reported heptanal as a biomarker of LC in 2005 [62], stating that while heptanal is less likely to be present in the EB of LC patients than hexanal, differences in heptanal concentrations between LC and HCs are significant to signify heptanal as a biomarker of LC [63]. Poli et al. reported a 13.9 pM median concentration of heptanal in the EB of LC patients compared to 6.1 pM in HCs, a significant difference that also identified heptanal as a biomarker of LC [8]. Corradi et al. was the only group to identify heptanal as a biomarker of LC in EB without also noting hexanal as a biomarker [78]. Deng et al. found heptanal in the EB of LC patients but not in the EB of HCs [61]. Three other reports deemed heptanal to be a biomarker of LC in EB: Handa et al. in 2014 [76] and Chen et al. [93] and Li et al. [95], both in 2021. Heptanal was reported in 39% of the papers collected for this review, and 53% of those studies considered heptanal to be biomarker of LC.

### 6.6. Octanal

Octanal is a possible LPO product of both ω-7 and ω-9 FAs. It was detected in the EB of LC patients in 36% of the papers collected for this study, 25% of which determined octanal to be a biomarker of LC. Fuchs et al. measured octanal as a biomarker in EB with a median concentration of 0.052 nmol/L in LC patients vs. a median concentration of 0.011 nmol/L in HCs [67]. Poli et al. reported similar results, with an octanal median concentration of 0.023 nmol/L in LC patients compared to a median concentration of 0.010 nmol/L in HCs [8]. Jouyban et al. reported an average concentration of octanal in the EB of LC patients to be 7.8 nmol/L, while HCs and patients undergoing treatment had levels lower than the LoD for the analytical method used; thus, they deemed octanal to be a biomarker of LC [87]. In 2021, Zou et al. also reported octanal as a biomarker of LC using a gradient boost decision trees algorithm on collected GC-MS data [97].

### 6.7. Nonanal

Nonanal, the third most detected aldehyde among the reports collected for this review, is formed by the LPO of ω-9 FAs. Fuchs et al. reported a median concentration of nonanal in the EB of LC patients of 0.239 nmol/L compared to a median concentration of 0.033 nmol/L in HCs [67]. Poli et al. reported somewhat lower nonanal median concentrations of 0.044 nmol/L in LC patients and 0.013 nmol/L in the EB of HCs [8]. Based on these results, both groups considered nonanal to be a biomarker of LC. Handa et al. not only reported nonanal as an EB biomarker of LC but also stated that its EB concentration can be used to distinguish between adenocarcinoma and squamous cell carcinoma [76]. More recently, Li et al. [95] and Long et al. [96] both identified nonanal as an EB biomarker of LC. Nonanal was reported in 41% of papers collected for this review, of which 28% determined nonanal is a biomarker of LC in EB.

### 6.8. Decanal

Decanal is an LPO product of ω-9 FAs. It was the least reported saturated aldehyde, both overall (20%) and as a biomarker when observed (22%). Schallschmidt et al. [83] and Long et al. [96] were the only groups to identify decanal as a biomarker of LC. Schallschmidt et al. reported a median concentration of decanal of 12.2 pmol/L in LC patients and 5.1 pmol/L in HCs. The high boiling point of decanal at 207 °C does require particular attention when establishing protocols for analysis by GC.

## 7. Unsaturated Aldehydes

### 7.1. 2-Propenal (Acrolein) and 2-Butenal (Crotonaldehyde)

2-Propenal is the most reactive α,β-unsaturated aldehyde because it is unsubstituted in the β-position. Consequently, 2-propenal readily disrupts cell functions due to facile reactions with biological nucleophiles, such as DNA, proteins, glutathione, and others [113]. 2-Butenal is similar to 2-propenal in terms of associated toxicity and also readily reacts with DNA and proteins [114]. Both aldehydes were detected in the EB of LC patients by Kischkel et al. [68], and 2-propenal was determined by Rudnicka et al. [69] to be a biomarker of LC. Whereas both of these aldehydes are known products of LPO, their merit as biomarkers is limited by the many other endogenous and exogenous sources [36,113,114]. In particular, among the largest contributing sources is smoking tobacco. Given that more than 88% of people with lung cancer recently surveyed were, or currently are, smokers [115], measurements of 2-propenal and 2-butenal in EB must be carefully considered in the context of patient history.

### 7.2. 2-Hexenal, 2-Heptenal and 2-Nonenal

The only reports of 2-hexenal, 2-heptenal, and 2-nonenal in the EB of LC patients come from Corradi et al. [78], who used a Bio-VOC tube for EB collection. This approach allowed for the targeted collection of alveolar breath, which helps to exclude many exogenous VOCs and environmental interferences. Only 4 of the 44 studies reviewed used Bio-VOC tubes for EB collection. Using this approach, Corradi et al. determined 2-nonenal to be a biomarker of LC.

### 7.3. 2-Decenal

2-Decenal was reported in association with LC only once, but not as a biomarker. Jouyban et al. detected aldehydes in the EB of LC patients by using a cold condensation tube and co-liquification protocol. As a result, they observed 2-decenal for the first time [87].

### 7.4. 4-Hydroxy-2-Hexenal (4-HHE)

4-HHE is a well-known product of LPO arising from the reaction of ω-3 FAs [116]. However, it has only been detected in the EB of LC patients by using one particular collection–analysis protocol, namely, derivatization to an oxime ether during preconcentration followed by analysis using FT-ICR-MS [56]. Fu et al. disclosed 4-HHE as a breath biomarker of LC and that 4-HHE concentration thresholds could be used to distinguish squamous cell carcinoma from adenocarcinoma and other NSCLCs [56]. Bousamra et al. also reported 4-HHE as a breath biomarker of LC and noted that after tumor resection, levels of 4-HHE in EB are significantly reduced and returned to levels found in HCs [74]. Li et al., in addition to reporting 4-HHE as an LC biomarker, reported that threshold concentrations of 4-HHE can be used to distinguish LC patients from patients with benign nodules (0.0073 nmol/L), smoking controls (0.0073 nmol/L), and non-smoking controls (0.0067 nmol/L) [79]. In 2015, Schumer et al. reported a median concentration of 4-HHE in HCs (0.001 nmol/L) compared to elevated concentrations of 4-HHE in early-(0.007 nmol/L) and late-stage cancer patients (0.009 nmol/L) [81]. In 2016, Schumer et al. also determined that the 4-HHE concentration in EB is reduced after tumor resection, reporting no significant difference between median concentrations in post-resection patients and HCs [84]. Of the papers collected for this review, only 11% observed 4-HHE in EB, but all those noted 4-HHE as a biomarker of LC. Though present in low concentrations, with proper preconcentration and analysis techniques, 4-HHE can be an excellent biomarker of LC due to its LPO origins and complete lack of environmental or other endogenous sources.

### 7.5. 4-Hydroxy-2-Nonenal (4-HNE)

4-HNE is derived from the LPO of ω-6 FAs [116]. Li et al. reported 4-HNE as a breath biomarker of LC, reporting threshold concentrations to distinguish LC from benign pulmonary nodules [79]. When comparing LC to patients with benign nodules, the threshold for LC is 0.00175 nmol/L, but when comparing LC to smoking controls or HCs, the thresholds are lower and at concentrations of 0.000285 and 0.000255 nmol/L, respectively [79]. In another study, Fu et al. observed significant differences in the 4-HNE concentrations that distinguish between SCLC and NSCLC in patients [56]. 4-HNE was reported in two of the papers collected for this review (5%), only one of which determined it to be a LC biomarker.

### 7.6. Malondialdehyde (MDA)

Tamura et al. [51] and Kawai et al. [52] both reported MDA as one of the many aldehydes produced during in vitro lipid peroxidation experiments, with its yield maximized when carried out at 37 °C [51]. In 2015, Li et al. disclosed the only report on MDA detected in the EB of LC patients but did not determine it as a biomarker [79]. In this study, MDA was detected by derivatization during preconcentration to a less reactive, cationic oxime ether analog, which may explain the ability of the researchers to detect this highly reactive enol-aldehyde. Interestingly, the large majority of studies reporting MDA in the EB of patients—patients with asthma [117], COPD [118,119], chronic airway inflammation [120], pulmonary disease [121], occupational hazard exposure [122,123,124,125], and air pollution exposure [126]—or in the EB from healthy subjects [127,128,129,130,131] relied on the chemical derivatization of MDA with either 2,4-dinitrophenylhydrazine (DNPH) or thiobarbituric acid (TBA) after the collection of exhaled breath condensate. Condensing MDA in this manner, converting it into more stable adducts, and then analyzing the adducts by LC-MS avoids the exposure of this highly reactive, thermally sensitive metabolite to heat. The thermal desorption step associated with SPME, the principal analytical technique employed in the Table 3 studies, likely precludes the detection of MDA, and possibly other unsaturated aldehyde metabolites, due to inducing reactions and/or decomposition.

## 8. Conclusions

Cancerous cells have increased metabolic activity and cellular dysfunction, leading to elevated levels of ROS. The excess ROS react with unsaturated lipids to form aldehyde metabolites via LPO. On considering the principal unsaturated fatty acids present in lung tissue and lung surfactant, it is reasonable to expect a panel of LPO-derived aldehydes consisting of saturated C3–C10 aldehydes, hydroxyaldehydes, and α,β-unsaturated aldehydes. This review examined all reports of volatile aldehydes in the EB of LC patients to summarize the efficacy of using the LPO-derived aldehyde panel as biomarkers of LC. The incidence of saturated aldehydes correlated often with LC, particularly in the case of pentanal, hexanal, and heptanal, which exhibited statistically significant elevations in concentration relative to HCs in near 50% of the studies that reported them. In contrast, there is a dearth of articles reporting hydroxyaldehydes or α,β-unsaturated aldehydes in the EB of LC patients, even though their formation via the random LPO process is also likely. 4-HHE was the most reported α,β-unsaturated aldehyde and was deemed a biomarker of LC 100% of the times it was detected. The studies reporting 4-HHE, as well as the other unsaturated biomarkers 2-nonenal and 4-HNE, all used chemical derivatization during preconcentration and analysis. The methods of preconcentration and analysis clearly impact not only the concentration ranges measured for the aldehyde metabolites but also which classes of aldehydes are detected. The quantification of reactive α,β-unsaturated aldehydes including MDA appears to require derivatization methods for accurate assessment as biomarkers.

Exhaled breath analysis is a rapidly growing field. In the two decades that followed the first reported aldehyde in LC patient breath, only four additional studies documented elevated levels of aldehydes in the EB of LC patients. Since then, however, there have been 39 studies on the EB of LC patients showing the merit of exhaled aldehydes as biomarkers. To fully realize the potential in using this class of LPO-derived metabolites as biomarkers of LC, the integration of chemoselective capture technology specific to aldehyde functionality with methods of analysis that take into account the sensitive nature of the more reactive aldehydes is needed.

## Figures and Tables

**Table 1:** Commonly reported FAs in lung tissue and lung surfactant [16,21].

Saturated FA		Monounsaturated FA (MUFA)		Polyunsaturated FA (PUFA)	
12:0 *^a^*	Lauric acid	16:1	Palmitoleic acid	18:2	Linoleic acid
14:0	Myristic acid	18:1	Oleic acid	18:3	Linolenic acid
16:0	Palmitic acid	20:1	Eicosenoic acid	20:2	Eicosadienoic acid
18:0	Stearic acid			20:3	Eicosatrienoic acid
				20:4	Arachidonic acid
				22:6	Docosahexaenoic acid

*^a^* XX:Y = number of carbons comprising the FA: number of double bonds in FA.

**Table 2:** Predicted LPO-derived aldehydes from a selection of unsaturated fatty acyl chains present in common ω-3 to ω-9 lung phosphatides.

Fatty Acid Sidechain	Aldehydes Predicted as LPO Products		
	Saturated	Unsaturated	Hydroxy
**ω-3** 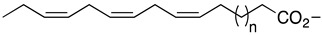 n = 5: α-linolenic acid (Δ^9,12,15^) n = 7: eicosatrienoic acid (Δ^11,14,17^)	propanal butanal	2-pentenal 2-hexenal	2-hydroxybutanal
		4-hydroxyhexenal (4-HHE)	
**ω-6** 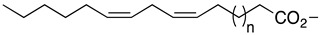 n = 5: linoleic acid (Δ^9,12^) n = 7: eicosadienoic acid (Δ^11,14^)	pentanal hexanal heptanal	2-octenal 2-nonenal	2-hydroxyheptanal
		4-hydroxynonenal (4-HNE)	
**ω-7** 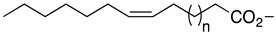 n = 5: palmitoleic (Δ^9^)	hexanal heptanal octanal	2-octenal	2-hydroxyheptanal
**ω-9** 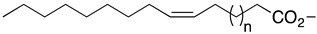 n = 5: oleic acid (Δ^9^) n = 7: eicosenoic acid (Δ^11^)	octanal nonanal decanal	2-decenal	2-hydroxynonanal

**Table 3:** Study details and exhaled aldehydes reported in breath analysis articles reviewed *^a^*.

Year	Study *^b^*	Patients *^c^*	Stage *^d^*	Breath Collection	Preconcentration Method	Analytical Instrument	Saturated Aldehydes	Unsaturated Aldehydes
1988	O’Neill [59]	8	NR	Teflon bag	Tenax TA	GC-MS	propanal, octanal, nonanal	
1999	Phillips [60]	108	I–IV	10 L collection apparatus	activated carbon	GC-MS	**hexanal**, **heptanal**	
2004	Deng [61]	10	I	sampling bulb	CAR/PDMS	GC-MS	**hexanal**, **heptanal**	
2005	Chen [62]	24	NR	Tedlar bag	SPME (unspecified)	GC-SAW sensor	**hexanal**, **heptanal**	
2007	Chen [63]	29	NR	Tedlar bag	PDMS	GC-FID	**hexanal**, **heptanal**	
2009	Bajtarevic [64]	285 *^e^*	NR	Tedlar bag	CAR/PDMS	PTR-MS/ GC-MS	**pentanal**	
2009	Gaspar [65]	18	IV	Tedlar bag	PDMS	GC-MS	hexanal, heptanal	
2009	Ligor [66]	65	NR	Tedlar bag	CAR/PDMS	GC-MS	pentanal	
2010	Fuchs [67]	12	III–IV	Tedlar bag	PDMS/DVB (PFBHA derivatization)	GC-MS	propanal, butanal, **pentanal**, **hexanal**, heptanal, **octanal**, **nonanal**, decanal	
2010	Kischkel [68]	31	II–IV	Tedlar bag	CAR/PDMS	GC-MS	**propanal**, butanal, pentanal, hexanal, heptanal, octanal	2-butenal
2010	Poli [8]	40	I–III	Bio-VOC tube	PDMS/DVB (PFBHA derivatization)	GC-MS	**propanal**, **butanal**, **pentanal**, **hexanal**, **heptanal**, **octanal**, **nonanal**	
2011	Rudnicka [69]	23	NR	Tedlar bag	CAR/PDMS	GC-MS	propanal, butanal, pentanal	
2011	Ulanowska [70]	137	NR	Tedlar bag	CAR/PDMS	GC-MS	**propanal**, **pentanal**, **hexanal**	
2011	Buszewski [71]	115	NR	Tedlar bag	CAR/PDMS	GC-MS	propanal, pentanal, hexanal	
2012	Buszewski [72]	29	NR	Tedlar bag	CAR/PDMS	GC-MS	propanal, **butanal**	
2012	Peled [73]	53	I–IV	Mylar bag	Tenax PA	GC-MS	decanal	
2014	Bousamra [74]	107	I–IV	Tedlar bag	Si microreactor (ATM derivatization)	FT-ICR-MS		**4-HHE**
2014	Filipiak [75]	36	NR	Tedlar bag	Tenax TA/CAR	GC-MS	butanal, pentanal, hexanal, nonanal, decanal	
2014	Fu [56]	97	I–IV	Tedlar bag	Si microreactor (ATM derivatization)	FT-ICR-MS	pentanal, hexanal, octanal, nonanal	**4-HHE**, 4-HNE
2014	Handa [76]	50	I–IV	—	expiration into spirometer	IMS	**hexanal**, **heptanal**, **nonanal**	
2014	Rudnicka [77]	108	I–IV	Tedlar bag	CAR/PDMS	GC-MS	propanal, pentanal, **hexanal**	
2015	Corradi [78]	71	I–IV	Bio-VOC tube	CAR/PDMS or PDMS/DVB (PFBHA derivatization)	GC-MS	propanal, butanal, pentanal, hexanal, **heptanal**, octanal, nonanal	2-hexenal, 2-heptenal, **2-nonenal**
2015	Li [79]	85	I–IV	Tedlar bag	Si microreactor (ATM derivatization)	FT-ICR-MS	**pentanal**	MDA, **4-HHE**, **4-HNE**
2015	Ligor [80]	123	III–IV	Tedlar bag	CAR/PDMS	GC-MS	propanal	
2015	Schumer [81]	156	0–IV	Tedlar bag	Si microreactor (ATM derivatization)	FT-ICR-MS		**4-HHE**
2016	Feinberg [82]	22	III–IV	QuinTron bag	aliquot *^f^*	PTR-MS	butanal, pentanal, hexanal	
2016	Schallschmidt [83]	37	NR	gas bulb and fleece tube	CAR/PDMS	GC-MS	**propanal**, **butanal**, pentanal, hexanal, heptanal, octanal, nonanal, **decanal**	
2016	Schumer [84]	31	0–IV	Tedlar bag	Si microreactor (ATM derivatization)	FT-ICR-MS		**4-HHE**
2016	Shehada [85]	149	I–IV	Tedlar bag	Tenax TA	Si nanowire sensor	**propanal**, **pentanal**	
2017	Callol-Sanchez [86]	81	I–IV	Bio-VOC tube	Tenax TA/graphitized carbon black/carbonized mol. sieve	GC-MS	hexanal, heptanal, **octanal**, nonanal	
2017	Jouyban [87]	7	IV	1 L glass sphere	breath condensate	GC-FID	hexanal, heptanal, octanal, decanal	2-decenal
2017	Sakumura [88]	107	I–IV	analytical barrier bag	breath condensate	GC-MS	nonanal	
2018	Wang [89]	233 *^g^*	NR	Tedlar bag	PDMS/Tenax TA	GC-MS	octanal, nonanal, decanal	
2019	Rudnicka [90]	108	I–IV	Tedlar bag	CAR/PDMS	GC-MS	propanal, pentanal, hexanal	
2020	Koureas [91]	51	NR	Tedlar bag	CAR/PDMS	GC-MS	hexanal, octanal, nonanal	
2020	Munoz-Lucas [92]	107	NR	Bio-VOC tube	Tenax TA/graphitized carbon black/carbonized mol. sieve	GC-MS	hexanal, heptanal, nonanal	
2021	Chen [93]	160	I–IV	Tedlar bag	Tenax TA	GC-MS	**hexanal**, **heptanal**	
2021	Gashimova [94]	40	I–IV	Tedlar bag	Tenax TA	e-nose sensor and GC-MS	butanal, **pentanal**, **hexanal**, heptanal, octanal, nonanal, decanal	
2021	Li [95]	6	NR	Tedlar bag	AgNP-coated chromatography paper	GC-MS	**propanal**, **butanal**, **pentanal**, **hexanal**, **heptanal**, octanal, **nonanal**, decanal	
2021	Long [96]	116	I–IV	Tedlar bag	DVB/CAR/PDMS	GC-MS	**nonanal**, **decanal**	
2021	Zou [97]	60	I–IV	Tedlar bar	Tenax TA	GC-MS	**octanal**	
2022	Larracy [98]	100	NR	—	Tenax TA	CRDS	hexanal	
2022	Soufi [99]	5	NR	Tedlar bag	POSS naphthalene diimine	GC-MS	pentanal, octanal, nonanal	
2022	Zou [100]	60	I–IV	Tedlar bag	Tenax TA	GC-MS	**hexanal**, octanal, nonanal	

*^a^* Aldehydes in **bold** were identified as biomarkers of LC, whereas aldehydes in normal typeface were detected but not directly correlated with LC; *^b^* first author of report and literature citation; *^c^* number of cancer patients examined; *^d^* lung cancer stage (NR = not reported); *^e^* 220 samples analyzed by PTR-MS, 65 samples analyzed by GC-MS; *^f^* an aliquot of the collected sample was removed for analysis, no preconcentration; *^g^* 108 samples were collected in Tedlar bags and preconcentrated by PDMS SPME, 125 samples were collected and preconcentrated using Tenax TA SPME.

## Abbreviations

**AgNP**	silver nanoparticle
**ATM**	2-aminooxy-*N*,*N*,*N*-trimethylethan-1-ammonium iodide
**CAR**	Carboxen
**CRDS**	cavity ring-down spectroscopy
**DNPH**	dinitrophenylhydrazine
**DVB**	divinylbenzene
**EB**	exhaled breath
**e-nose**	electronic nose
**FA**	fatty acid
**FT-ICR-MS**	Fourier-transform ion cyclotron resonance mass spectrometry
**GC-MS**	gas chromatography-mass spectrometry
**HC**	healthy control
**4-HHE**	4-hydroxy-2-hexenal
**4-HNE**	4-hydroxy-2-nonenal
**IMS**	ion mobility spectrometry
**LC**	lung cancer
**LoD**	limit of detection
**LPO**	lipid peroxidation
**MDA**	malondialdehyde
**MUFA**	monounsaturated fatty acid
**NSCLC**	non-small cell lung cancer
**NR**	not reported
**OS**	oxidative stress
**PC**	phosphatidylcholine
**PDMS**	polydimethylsiloxane
**PE**	phosphatidylethanolamine
**PFBHA**	(pentafluorobenzyl)hydroxylamine
**PG**	phosphatidylglycerol
**PL**	phospholipid
**POSS**	polyhedral oligomeric silsesquioxane
**PS**	phosphatidylserine
**PTR-MS**	proton transfer reaction-mass spectrometry
**PUFA**	polyunsaturated fatty acid
**ROS**	reactive oxygen species
**SAW**	surface acoustic wave
**SCLC**	small cell lung cancer
**Sph**	sphingomyelin
**SPME**	solid-phase microextraction
**TBA**	thiobarbituric acid
**VOC**	volatile organic compound
